# Chenodeoxycholic Acid Pharmacology in Biotechnology and Transplantable Pharmaceutical Applications for Tissue Delivery: An Acute Preclinical Study

**DOI:** 10.3390/cells10092437

**Published:** 2021-09-16

**Authors:** Armin Mooranian, Corina Mihaela Ionescu, Susbin Raj Wagle, Bozica Kovacevic, Daniel Walker, Melissa Jones, Jacqueline Chester, Edan Johnston, Maja Danic, Momir Mikov, Crispin Dass, Hani Al-Salami

**Affiliations:** 1 Biotechnology and Drug Development Research Laboratory, Curtin Medical School, Health Innovation Research Institute, Curtin University, Perth 6102, Australia; A.Mooranian@curtin.edu.au (A.M.); c.ionescu@postgrad.curtin.edu.au (C.M.I.); susbinraj.wagle@postgrad.curtin.edu.au (S.R.W.); bozica.kovacevic@postgrad.curtin.edu.au (B.K.); danieljcswalker@gmail.com (D.W.); melissa.a.jones@postgrad.curtin.edu.au (M.J.); j.chester@student.curtin.edu.au (J.C.); edan.johnston@student.curtin.edu.au (E.J.); 2 Hearing Therapeutics, Ear Science Institute Australia, Queen Elizabeth II Medical Centre, Perth 6009, Australia; 3 Department of Pharmacology, Toxicology and Clinical Pharmacology, Faculty of Medicine, University of Novi Sad, 21101 Novi Sad, Serbia; majadjanic@gmail.com (M.D.); mikovmomir@gmail.com (M.M.); 4 Curtin Medical School & Curtin Health Innovation Research Institute, Curtin University, Perth 6102, Australia; crispin.dass@curtin.edu.au

**Keywords:** chenodeoxycholic acid, primary human bile acid, transplantation, type 1 diabetes

## Abstract

Introduction. Primary bile acids (PBAs) are produced and released into human gut as a result of cholesterol catabolism in the liver. A predominant PBA is chenodeoxycholic acid (CDCA), which in a recent study in our laboratory, showed significant excipient-stabilizing effects on microcapsules carrying insulinoma β-cells, in vitro, resulting in improved cell functions and insulin release, in the hyperglycemic state. Hence, this study aimed to investigate the applications of CDCA in bio-encapsulation and transplantation of primary healthy viable islets, preclinically, in type 1 diabetes. Methods. Healthy islets were harvested from balb/c mice, encapsulated in CDCA microcapsules, and transplanted into the epididymal tissues of 6 syngeneic diabetic mice, post diabetes confirmation. Pre-transplantation, the microcapsules’ morphology, size, CDCA-deep layer distribution, and physical features such as swelling ratio and mechanical strength were analyzed. Post-transplantation, animals’ weight, bile acids’, and proinflammatory biomarkers’ concentrations were analyzed. The control group was diabetic mice that were transplanted encapsulated islets (without PBA). Results and Conclusion. Islet encapsulation by PBA microcapsules did not compromise the microcapsules’ morphology or features. Furthermore, the PBA-graft performed better in terms of glycemic control and resulted in modulation of the bile acid profile in the brain. This is suggestive that the improved glycemic control was mediated via brain-related effects. However, the improvement in graft insulin delivery and glycemic control was short-term.

## 1. Introduction

Primary bile acids (PBAs) are produced in human gut, and result from cholesterol breakdown by hepatocytes, before being released from the liver and stored in the gall bladder and secreted into the intestine upon food ingestion [1]. In the intestine, the human microbiome metabolizes PBAs into secondary bile acids, which are then reabsorbed back into the liver for further metabolism, resulting in a pool of primary, secondary, and tertiary bile acids, also known as the bile acid pool [2]. Based on the literature, the potency profile of these bile acids follows the order of ursodeoxycholic acid (UDCA) < chenodeoxycholic acid (CDCA) < lithocholic acid (LCA), with UDCA being the least potent or toxic [3,4,5]. 

A dominant PBA in the gut is CDCA, which, of recently, has been shown to exert beneficial effects in formulating drug matrices, as an excipient and as a matrix stabilizing agent. In a recent study, when incorporated with matrix formulation, CDCA showed excipient-stabilizing effects on microcapsules carrying viable insulinoma β-cells, in vitro [6]. In another study in our laboratory, the incorporation of CDCA improved the stability and reduced swelling of drug-loaded microcapsules resulting in improved drug stability and release and overall better shelf-life [7]. In addition to its potential applications as a stabilizing excipient in formulations, CDCA and other bile acids have also been shown to exert significant pharmacological and biological effects. 

Based on published studies, when insulin sensitivity was correlated with the bile acid pool, there was a strong association between bile acids’ ratios and glucose tissue uptake, suggesting that bile acids may be directly related to glucose regulation and cellular uptake [8]. In a study by Cariou et al., fasting plasma concentrations of CDCA as well as other bile acids were found to be inversely correlated with insulin sensitivity in human healthy and diabetic adults. The authors proposed the mechanisms to be at the molecular levels, affecting a wide range of nuclear receptors, including farnesoid X-receptor (FXR) [9]. In another study by Shihabudeen et al., the authors found that CDCA can be used to treat liver cirrhosis due to its role in suppressing inflammatory regulators, reversing insulin resistance, and modulating secretion of pro-inflammatory and anti-inflammatory adipokines [10]. Other studies have shown that in addition to its beneficial biological effects in liver disease, CDCA possesses wide pharmacological effects but potentially might not be as potent as other bile acids such as UDCA [11,12,13]. UDCA has been shown to possess widespread anti-inflammatory and anti-apoptotic effects and can also exert beneficial effects on blood glucose profile via reduced gluconeogenesis, increased insulin sensitivity, and energy expenditure [12,13,14,15]. On the other hand, it is worth stating that not all bile acids are known for their beneficial biological and pharmacological effects. For example, the bile acid LCA can be toxic and is often attributed to inflammation, tissue necrosis, as well as cancer development [11,12,16]. 

Type 1 diabetes is a chronic condition which presents with insulin deficiency which causes subsequent hyperglycemia. Type 1 diabetes also presents with a range of complications which results in the condition often being difficult to treat and manage. Simplistically, insulin treatment is required for type 1 diabetic patients. Such insulin treatments have historically been administered via injection, with a stringent multiple-dose regiment required in order to closely mimic a functional physiological insulin level. Over time, alternative insulin analogues have been developed in an attempt to improve insulin delivery and uptake. An insulin pump style delivery has also been implemented to allow a continuous treatment with insulin [17,18]. Whilst these strategies have been effective at maintaining insulin levels, the removal of the necessity of such injectable insulin therapies would be greatly beneficial to the treatment of type 1 diabetes. A bioartificial pancreas has been proposed, with islet transplantation proposed to be most successful via the microencapsulation for the immunoisolation of islets, as investigated by several preclinical trials [19,20]. This includes a study by Dufrane et al. with microencapsulated pig islets with polymer sodium alginate transplanted to primates, with partial islet survival for up to six months without immunosuppressants [21]. The potential for treatment with transplanted islets is greatly advantageous, removing the requirement for exogenous insulin therapy and removing many of the complications which are associated with type 1 diabetes due to the implantation of functional islets without the requirement for immunosuppressive medications associated with traditional orthotopic pancreatic transplant [22,23].

In terms of the relationships between bile acids, glucose, insulin, and diabetes, several studies have shown interesting results. Of these, multiple investigated bile acid impacts on the liver and subsequent results in glucose homeostasis. Seyer et al. showed bile acids or FXR agonistic treatment of islets to result in an increase in insulin secretion stimulated by glucose, with overall results demonstrating that bile acids may influence β-cell glucose competence in the liver [24]. Other studies suggest that in human hepatocytes, CDCA can regulate the synthesis of bile acids without the need for fibroblast growth factor 19, which is known to regulate bile acid homeostasis [25]. Diet has also been suggested to moderately impact bile acids, which may also affect glucose homeostasis [26]. Bile acid signaling is also shown to be expressed in colonic enteroendocrine cells which are deficient in obese and diabetic patients, impacting glucose homeostasis [27]. Other studies which may be of interest include the following references [28,29].

Overall, in the context of drug and islet formulation and delivery, CDCA seems to have strong effects on stabilizing microcapsules containing islets, as well as exerting positive biological effects on islets that may improve their ability to survival and function, post-transplantation [6,7]. Accordingly, in this study, in order to investigate the applications and potential role of CDCA in islet transplantation, healthy islets were harvested from mice, encapsulated in CDCA microcapsules, and surgically transplanted into the epididymal tissues in pelvic region of 6 syngeneic diabetic mice (injected with alloxan to induce T1D, confirmed with blood glucose >16 mM in two consecutive measurements over two days, and absence insulin in blood). The CDCA was used to complement the stability of the delivery system, and the amounts used were pharmacologically negligible. The effects of CDCA incorporation on microcapsule morphology and formulation characteristics were assessed, and CDCA microcapsules were evaluated for the size, elemental composition, CDCA-surface distribution, and physical features. Upon transplantation, the survival rate, inflammation profile, concentrations of bile acids, and pro-inflammatory biomarkers in biological samples of T1D animal model were assessed post-transplantation.

## 2. Methods

### 2.1. Materials

Calcium and barium chloride were acquired from Scharlab S.L (Barcelona, Spain), and PBA CDCA, poly-L-ornithine, alginate sodium, and mixing reagents were purchased from Sigma Chemical Co. (St. Louise, MO, USA) and Thermo Fisher (Scoresby, VIC, Australia). Control and test microcapsules were prepared using our Ionic Gelation Vibrational Jet Flow technology [30,31,32,33,34,35,36,37,38]. Formulation excipients consisted of 2% PBA, 2% CDCA, 1.5% poly-l-ornithine, and 1.8% sodium alginate in 1% gel. The matrices were formulated within 48 h prior to islet encapsulation and surgical transplantation. All formulations were stored in the refrigerator when not in use, and were used within 72 h of preparation. 

### 2.2. Islet Microencapsulation, Topographic, Size Distribution, Surface Elemental Composition, and Bile Acid Distribution Profiles, and Swelling and Mechanical Property Ratios Assessments

The microencapsulation of islets was performed under sterile conditions as per our established methods in pancreatic cell encapsulation [39,40,41,42,43,44,45,46,47,48]. The effect of CDCA incorporation on islet-containing microcapsules were analyzed in terms of topographic features, spectral elemental composition, microcapsule-size distribution, CDCA distribution on the surface of the microcapsules, and microcapsules’ swelling and mechanical strength profiles. All measurements were carried out based on our well-established methods [40,41,42,43,44,45,46,47,48,49,50,51].

Briefly, CDCA microcapsules were prepared using our Ionic Gelation Vibrational Jet Flow technology via ionotropic gelation processed using main encapsulating parameters, based on Büchi customized technology (Büchi, Switzerland) [30,40,52,53,54,55,56,57,58,59]. A multitude of topographic assessments were conducted, with microscopy imaging, surface spectral analyses and CDCA surface distribution assessments, scanning electron imaging, energy dispersive X-ray spectroscopy, and confocal image measurements; all of which were carried out on three randomly selected batches at the John De Laeter center, as well as at the Curtin Health Innovation Research Institute (Bentley, WA, Australia). Zeiss Neon 40EsB FIBSEM (Oberkochen, Germany), Oxford Instruments Aztec X-Act (Abingdon, UK), Olympus IX-51 and Nikon A1 confocal system (Tokyo, Japan) were used. For topography and surface elemental composition analyses, microcapsules were coated with platinum, dried, and analyzed using laser-guided imagining. For CDCA surface distribution, CDCA-conjugate was prepared and imaged using confocal-Nikon surface imaging, as per our well-described techniques [45,60,61].

Size distribution, swelling, and mechanical resistance ratio measurements were carried out using our well-established methods [44,45,50,62]. The size distribution of the microcapsules was assessed using Master Sizer 2000 (Malvern, UK), while swelling ratio and mechanical resistance were assessed using our methods of weight loss and structure integrity assessments [49,63]. Briefly, 50 islet-containing microcapsules were incubated in phosphate buffer at 37 °C and after one week of incubation, the swelling resistance index was determined by comparing the initial and final weight of microcapsules [36,64]. The percentage of intact microcapsules was calculated to determine the swelling resistance index. The mechanical strength was investigated by placing the microcapsules in phosphate buffer and subject to the external mechanical agitation and disturbances over one week using Boeco Shaker (Hamburg, Germany).

Once all microencapsulation and characterization assessments were carried out, the preclinical studies commenced.

### 2.3. Preclinical Study Design

All experiments were approved by the Animal Ethics Committee at Curtin University and all experiments were performed in accordance with the Australian Code of Practice for the care and use of animals for scientific purposes.

Mice were acclimatized for up to one week after their arrival at the animal holding facility at Curtin University as per normal protocols. The study design and preclinical investigation encompassed two equal groups of mice, induced with diabetes (alloxan; 150 mg/Kg; IP/SC) and once diabetes confirmed (blood glucose >16 mM in two consecutive days, and absence of plasma insulin), both groups transplanted viable islets, harvested from donor healthy syngeneic mice. Diabetes induction and confirmation were carried out using our well-established methods [31,34,58,59,65,66].

**Figure 1:**
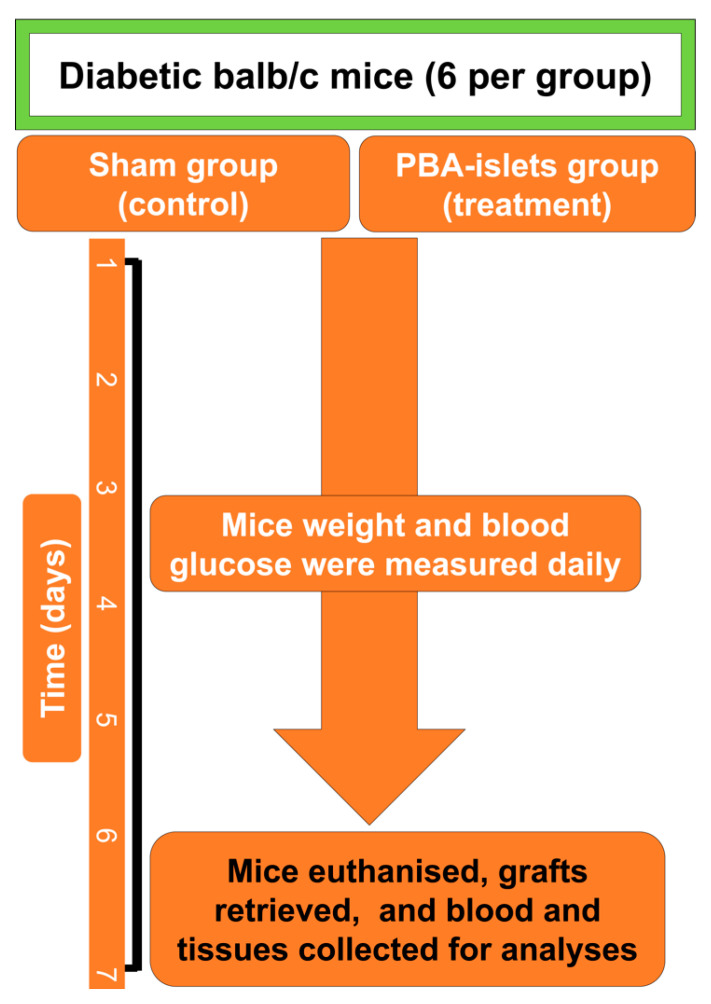
Experimental design and timeline for the surgical transplantation of islet-loaded CDCA microcapsules.

For the donor mice, they were euthanized and their islets harvested, digested, and encapsulated before being transplanted into both recipient groups of mice. Group-1 mice were considered control and were transplanted encapsulated viable islets. Group-2 mice were considered treatment and were transplanted CDCA-encapsulated viable islets. Both recipient groups as well as the donor mice were syngeneic, adult male balb/c, 6–8 weeks old. Harvested islets were encapsulated using our well-established methods [61] and transplanted surgically into the epididymal tissues, within 24 h from being harvested. The experiment duration was 7 days, following which mice were euthanized and blood, tissues, and feces were collected for analysis. Two main sets of measurements were carried out. The first set on the topographic and physical and pharmaceutical features of the CDCA-microcapsules and the other set for the biological and pharmacological effects of the transplanted CDCA-microcapsules containing viable islets [67] (Figure 1).

### 2.4. CDCA-Islet Epididymal Surgical Transplantation

**Figure 2:**
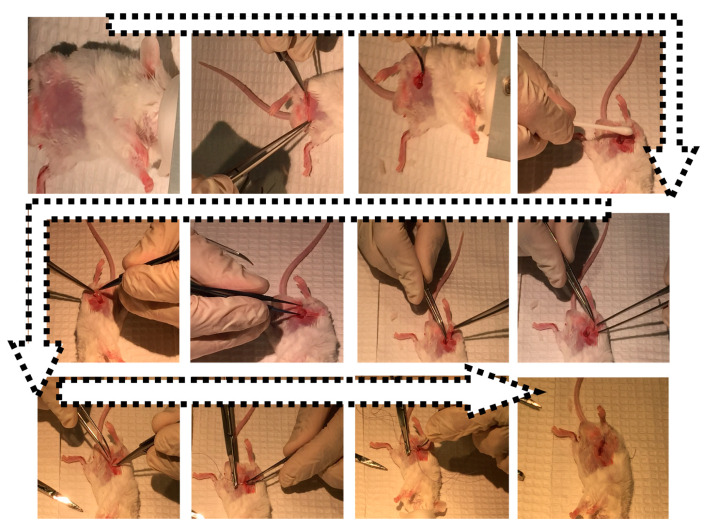
Surgical steps in the epididymal transplantation of encapsulated islets using CDCA-based microcapsules (an illustration surgical procedure).

Recipient mice were transplanted donor mice islets, encapsulated in formulation matrix (control) or CDCA (test). Islet extraction from donor mice was carried out as per our in-house developed and established protocols that have been approved by the Animal Ethics Committee at Curtin University. Upon euthanasia, 3 mL of collagenase in RPMI media was injected into the pancreatic duct to isolate the pancreas. The pancreas was inflated by incubation in a water bath at 37 °C for 15 min before being vortexed at 2500 rpm, and the suspension was retrieved by filtration into a 50-mL tube. The supernatant was discarded by successive vortexing and centrifugation, followed by collection of islets using a serological pipette customized for islet collection. The epididymal surgical transplantation of encapsulated islets in both recipient groups was carried out as per approved protocol and antibiotics were applied to prevent potential infection (Figure 2). Animals were monitored pre- and post-surgery as per our approved protocols. Appropriate heating pads, special surgical housing, soft food, and easy water access were provided to ensure the best animal welfare was maintained. Further applications of opioid pain killers or antibiotics to mice were carried out as per approved conditions in order to ensure the best outcome. Surgical complications were monitored such as swelling or bleeding, in order to ensure robust scientific data.

### 2.5. Assessments of the Inflammatory and the Bile Acid Profiles

In order to assess the inflammatory profile, the proinflammatory cytokine, interleukin-6 (IL-6) was measured in plasma using BD Biosciences CBA technology (San Jose, CA, USA) as per our well-established methods [61,68,69]. In order to assess the bile acid profile, concentrations of the bile acids CDCA, LCA, and UDCA were analyzed in blood, tissues, and feces. The three bile acids were measured in plasma, brain, liver and feces of recipient mice in both control and treatment groups using our well-established liquid chromatography mass spectrometry (LCMS 2020 system, Shimadzu Corporation, Japan) system, according to the established protocols [69,70,71]. In order to extract the aforementioned bile acids from the plasma, tissues, and feces, the samples were mixed with acetonitrile at a ratio of 1:1. After centrifugation of the samples, 10 µL of supernatant was injected into the LCMS system. The bile acids were separated by a C-18 column with 5-µm pore size (Phenomenex, Torrance, CA, USA), and a mobile phase that was composed of methanol and water at a ratio of 65%:35%.

### 2.6. Statistical Analysis

Statistical analysis was conducted using Prism^®^ software v.9 (GraphPad Software, Inc., La Jolla, CA, USA), with one-way ANOVA being the analysis technique of choice. *p* < 0.05 was used for statistical significance. 

## 3. Results

### 3.1. Topographic Features, Size Distribution, Surface Elemental Composition, Chenodeoxycholic Acid Distribution, and Swelling and Mechanical Property Measurements

**Figure 3:**
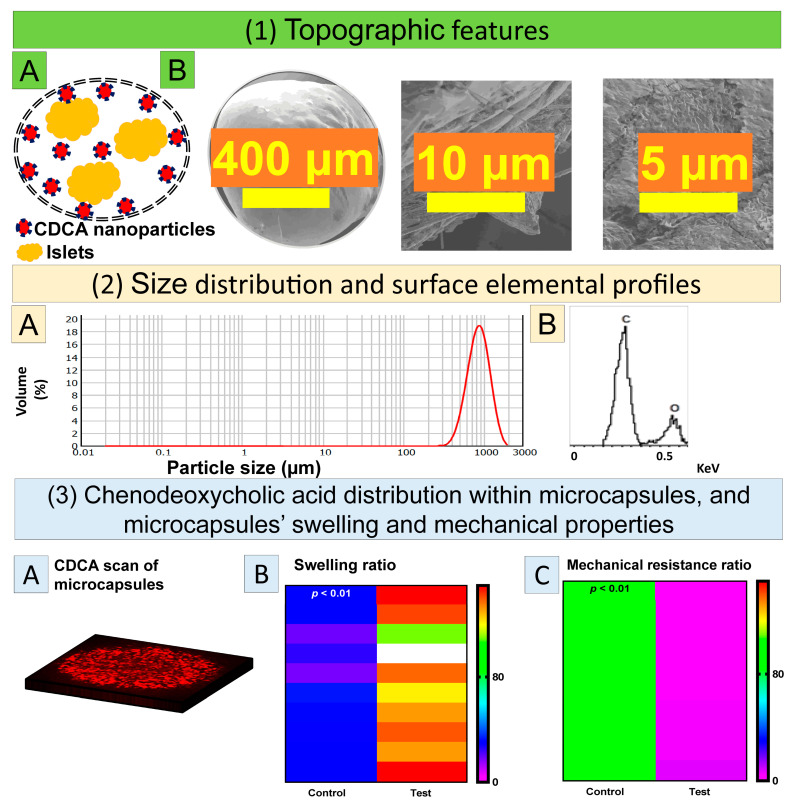
Schematic diagram (**1A**), SEM micrographs (**1B**), size distribution (**2A**), EDS analysis (**2B**), confocal assessment of CDCA distribution within microcapsules (**3A**), swelling (**3B**) and mechanical index (**3C**) of CDCA microcapsules.

Figure 3 shows the schematic diagram (1A), SEM micrographs (1B), size distribution (2A), EDXS analysis (2B), confocal assessment of CDCA distribution (3A), swelling (3B) and mechanical resistance (3C) of the islet-loaded CDCA microcapsules. 

When using our Ionic Gelation Vibrational Jet Flow technology to fabricate microcapsules, the incorporation of islets within the microcapsules resulted in spherical shape microcapsule (Figure 3(1B)) of uniform size (Figure 3(2A)) with the surface elemental chemical composition representative of the nature of the polymers and excipients used. There was uniform and visible CDCA distribution within the matrix of the microcapsules (Figure 3(3A)) and microcapsules displayed robust resistance to osmotic induced swelling and mechanical degradation (Figure 3(3B,C)). Therefore, the results of this section have shown the co-encapsulation of exogenous CDCA with islets, when compared to controls without CDCA, to have consistent chemical composition and resistance to osmotic stress, improving mechanical strength. 

**Figure 4:**
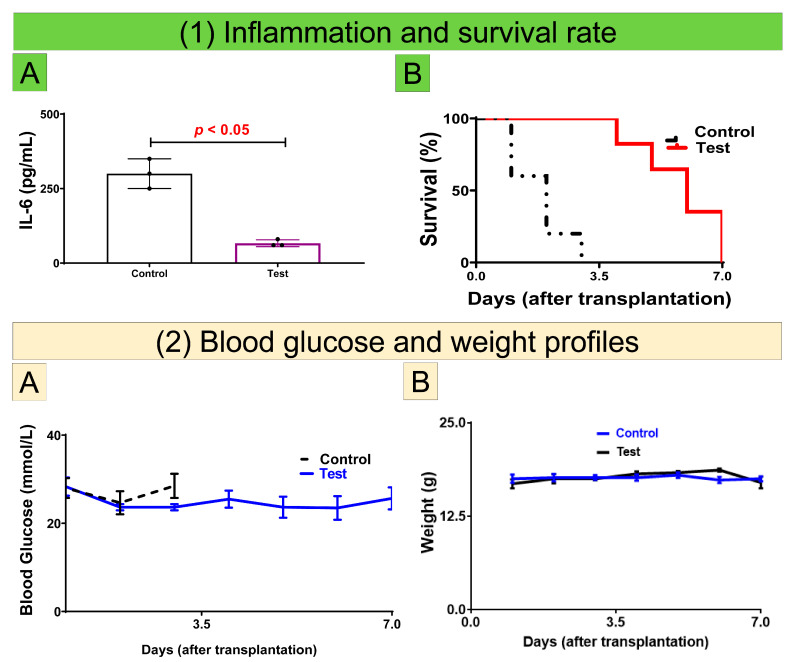
IL-6 plasma levels (**1A**), survival (**1B**), blood glucose (**2A**), and weight (**2B**) of transplanted mice. Data are average ± SEM. Sample size N = 6.

Figure 4 shows plasma levels of the proinflammatory biomarker, IL-6 (Figure 4(1A)), survival rate (Figure 4(1B)), blood glucose (Figure 4(2A)), and weight (Figure 4(2B)) of transplanted mice. As it can be seen, mice transplanted with islets-loaded microcapsules survived for several days longer than the control (non-CDCA microcapsules) group (Figure 4(1B)) as well as displayed improved blood glucose levels (Figure 4(2A)), which was complemented with corresponding decreases in the plasma levels of the pro-inflammatory cytokine IL-6 (Figure 4(1A)). As can be seen in Figure 4(2B), both the control and test showed results of similar weight profiles. The amounts of pro-inflammatory cytokine IL-6 present in the plasma of recipient mice treated with CDCA-islet microcapsules were more than 60% lower than the group treated with microencapsulated islets, suggesting an immune-protective effects of CDCA. 

**Figure 5:**
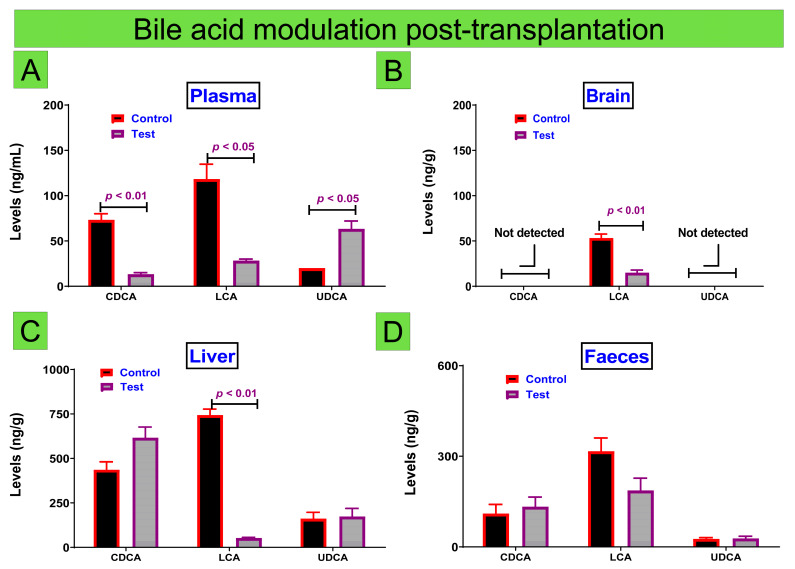
Bile acid levels in control (red) and test transplanted mice (purple) showing data for plasma (**A**), brain (**B**), liver (**C**), and feces (**D**). Data are mean ± SEM. Sample size N = 6.

Recent studies have demonstrated significant effects of diabetes induction, development, and progression on the bile acid profile [55] and hence, it is likely that the positive glycemic and antidiabetic effects by the transplanted CDCA-islet microcapsules may modulate the bile acid profile in these treated mice, compared with control (Figure 5).

### 3.2. The Bile Acid Profile and Diabetes Treatment

Figure 5 shows the levels of endogenous bile acids (CDCA, LCA, and UDCA) in plasma (Figure 5A), brain (Figure 5B), liver (Figure 5C), and feces (Figure 5D) in both groups of mice: control and test.

In plasma, treatment caused significant reduction in CDCA and LCA levels and an increase in UDCA levels, compared with the control. The reduction in CDCA suggests either reduction in cholesterol catabolism and CDCA synthesis, or an increase in CDCA gut metabolism and subsequent CDCA reduction in concentration. The reduction in LCA suggests either accelerated metabolism of LCA by the gut microbiome or a reduction in PBA metabolism that results in a reduction in synthesis of LCA. Accordingly, decreased levels of CDCA in plasma of our treated mice compared with the control (Figure 5A) suggest that CDCA-islet microcapsules exacerbated the reduction in cholesterol catabolism or increased CDCA metabolism by gut microbiome. The LCA reduction appears to be associated with reduced inflammation due to the toxic nature of LCA, while UDCA induction seems to associate with positive glycemic control and improved inflammatory profile. In the brain, treatment caused a significant reduction of LCA with no detected levels of CDCA or UDCA. In the literature, studies have shown that CDCA intake caused increased levels of UDCA and that was as a result of the upregulation of UDCA synthetic pathways and modulation in the bile acid profile [72]; while other studies have demonstrated the presence of multiple metabolites and intermediates that mediate biosynthesis of primary bile acids such as CDCA in tissues [73]. The absence of CDCA and UDCA in brain of diabetic mice suggests a reduced bile acid profile within brain tissues of diabetic mice, while the presence of LCA, which was reduced by treatment, suggests the reduction is due to the reduced inflammation reported in Figure 4(1A), since inflammation has been closely associated with LCA levels in plasma [74,75,76]. In the liver, treatment caused a significant reduction in LCA levels whilst there was no significant alteration to the CDCA or UDCA levels. Although the increase of CDCA levels due to treatment did not reach significance, it remains visible, which might be due to the fact that since the liver is the site where CDCA is synthesized, higher CDCA levels are caused by an overall reduction in inflammation and improved glycemia and hence, better blood circulation resulting in more efficient cholesterol catabolism and production of CDCA. On the other hand, the significant and substantial reduction in LCA levels in the liver may be due to overall reduced inflammation and improved glycemia resulting in normalization of LCA levels in the treated diabetic mice (Figure 5C). Moreover, Figure 5C shows that UDCA levels remain similar among control and treatment groups suggesting lack of direct association between CDCA-islet transplantation and bile acid synthesis in the liver and metabolism in the gut over the duration of the experiment. In feces (Figure 5D), treatment did not cause significant and substantial alteration to excreted bile acids. Given that the enterohepatic recirculation of bile acids account for more than 90% of total bile acids, lack of significant alteration due to CDCA-islet transplantation was somewhat expected. The effects of CDCA on islet biology have previously been reported by our group [6,38,77].

## 4. Discussion

The field of islet transplantation to treat type 1 diabetes is well established and research has been ongoing for many years [78]. The research aimed to replace injectable insulin as a way to revolutionize diabetes treatment.

Since its discovery in 1921, injectable insulin remains the mainstream treatment for treating type 1 diabetes and although effective, its route of administration remains problematic in terms of patient compliance, injection complications, and storage challenges. Researchers have invested significant funding and time in order to revolutionize injectable insulin and various attempts have been trialed including design of interactive automated hybrid systems that consistently measure glucose and inject insulin, design of new stable insulin mimetics, design of new nanocapsules for oral, nasal, or pulmonary delivery of insulin, and design of new hydrogels suitable for islet delivery and transplantation, with the ultimate goal of complementing or even replacing the need for injectable insulin [79,80,81]. However, and despite the best effort and ongoing research, an ultimate treatment replacing injectable insulin has not been established in the clinic and wide applications of islet transplantation as a method to replace insulin therapy, long-term, have not been successful or commercially viable. Alternative inventions for designing better insulin delivery systems also failed to meet the clinical need to treating Type 1 diabetes, and hence, better approaches remain to be achieved for such a medical need. Hence, this study aimed to explore the applications of primary bile acids (PBAs) in islet transplantation, and insulin delivery with a particular interest in the PBA, CDCA.

In terms of the surface elemental composition of the microcapsules, the results demonstrated such composition to be characteristic of the encapsulation polymers and excipients which make up the microcapsules. Therefore, these results were in accordance with published studies demonstrating that bile acid incorporation with microcapsules did not compromise the nature of excipients of these microcapsules and atoms such as C and O remain integral to the surface characteristics of these microcapsules (Figure 3(2B)) [6,42,44]. Furthermore, the microcapsules were shown to be resistant to any osmotic induced swelling and mechanical degradation, with the findings being consistent with the literature. Thus, the current study reveals that the CDCA microcapsules could effectively co-encapsulate exogenous CDCA and islets for transplantation, in addition to superior stability against osmotic and mechanical stress, when compared with non-CDCA encapsulated islets. Accordingly, CDCA-islet microcapsules exhibit features that may promote better islet survival, glycemic control, and reduced inflammation (Figure 4).

As mentioned in the results section, pro-inflammatory cytokine IL-6 measurements in plasma of mice had significant differences when comparing CDCA microencapsulated islets to non-CDCA microencapsulated islets. This is consistent with previous studies that showed immune-protective effects of bile acids when incorporated with nano and microcapsules and exposed to cells either in cell culture or in a preclinical setting [46,49,82]. Accordingly, findings suggest that CDCA incorporation with islets have direct biological effects on islet functions, insulin release, and glycemic response, and can also exert significant anti-inflammatory effects, potentially further improving the islets insulin release and diabetes treatment. Such desirable biological and anti-inflammatory effects is likely to result in improved overall survival rate of transplanted graft and the host. Insulin levels after transplantation averaged slightly lowering than normal levels seen in healthy mice, but large enough to exert pharmacological effects.

For the weight profiles comparing non-CDCA transplanted islets and CDCA transplanted islets, similar weight profiles, (Figure 4(2B)), suggest that the improved glycemia and inflammatory profiles are not directly the result of weight gain or improved weight profile, but rather improved glycemic control due to better tissue and cell viability and better insulin release from the CDCA-islet microcapsules, compared with control. This is consistent with the literature, which has demonstrated significant pharmacological and endocrinological effects of bile acids in cell signaling and functions, and overall viability and biological activities [2].

Bile acid production and metabolism are complex and multifaceted. Endogenous bile acids such as CDCA are produced via cholesterol catabolism. They are metabolized by the gut microbiome into, for example, LCA and UDCA, and are recycled multiple times a day, through what is known as the enterohepatic recirculation pathways. There are many different types of bile acids that present in gut and various parts of the body, and their synthesis is regulated by feedback mechanisms and complex processes [83,84,85]. In this study, the impact of transplanting CDCA-islet microcapsules on the bile acid pool is likely to be caused mainly as the result of the biological effects (including glycemic and inflammatory effects) rather than feedback mechanisms brought about directly and predominantly due to the presence of CDCA in the body, within the transplanted microcapsule.

Previously published studies in type 1 diabetes development and the bile acid profile reported significant reduction in plasma CDCA levels and increase in plasma LCA levels demonstrating negative feedback mechanisms on CDCA levels and positive feedback mechanisms on LCA levels as a result of diabetes development, while UDCA levels were not significantly altered [55]. Other studies have shown that induction of type 2 diabetes resulted in a decrease in UDCA levels in plasma, demonstrating negative feedback mechanisms as a result of diabetes development [49]. Furthermore, it has been shown that changes in the bile acid pool may be observed prior to type 2 diabetes development, suggesting that bile acids may play a role [86]. Type 1 diabetes findings were consistent with this research, which demonstrated, compared to the control, a significant reduction in CDCA and LCA levels in plasma, whilst there was an increase in plasma UDCA levels. The reduction in LCA plasma appearing to associate with reduced inflammation and the increase in plasma UDCA is likely associated with both positive glycemic control and inflammatory profile improvement. Such results are consistent with previously published studies that demonstrated positive UDCA effects on the inflammatory biomarkers [39,87].

Changes were observed in the bile acid pools, although there was no significance in the changes between UDCA and CDCA. Statistically insignificant changes in CDCA were observed, with increases in levels likely to be due to improved blood circulation. Decreases in LCA were statistically significant in the liver, likely to be due to overall inflammation reduction and glycemia reduction. Published studies suggest that provision of certain bile acids can result in alteration in the bile acid profile via vitamin D receptors. In a study carried out by Nishida et al., the authors showed that administration of CDCA resulted in alteration of the bile acid profile, particularly the bile acid LCA, via direct influence on vitamin D receptors and LCA metabolism pathways [88]. Other studies in our laboratory have suggested that diabetes induction has been associated with increased LCA levels in tissues and feces due to a potential increased expressions of liver enzymes and nuclear receptors as well as alteration in the bile acid enterohepatic recirculation processes causing a shift in the bile acid hemostasis and subsequent increase in LCA synthetic pathways [55].

## 5. Conclusions

**Figure 6:**
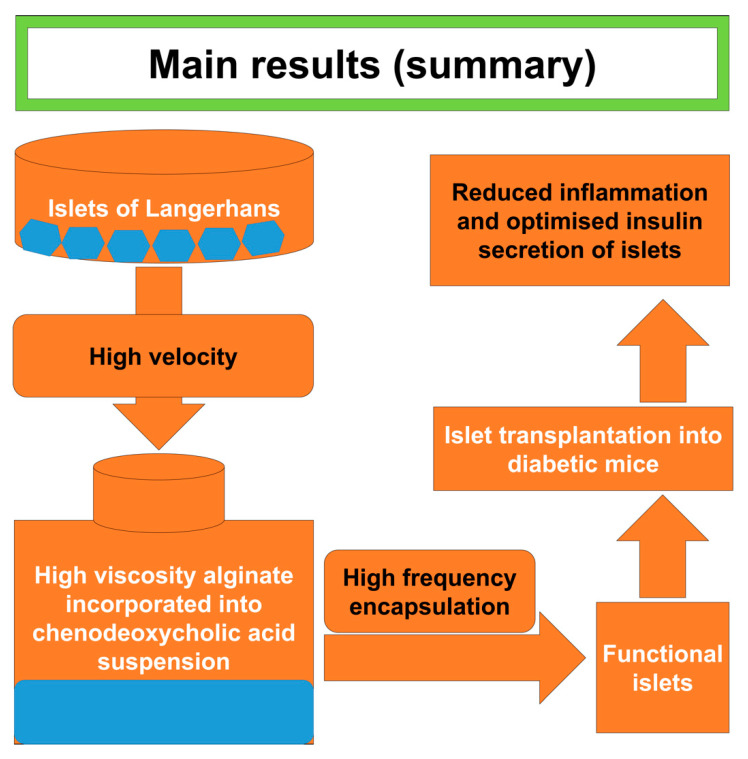
Overview of the results produced from this study.

The study has investigated the effects of CDCA incorporation on microcapsule formation and primary islet encapsulation. The results showed that CDCA incorporation into islets containing microcapsules enhanced the integrity and stability of the microcapsules. In the presence of CDCA, post-transplantation, the encapsulated islets showed improved biological effects, including desirable islet functions, insulin release and glycemic response. Furthermore, the incorporation of CDCA reduced inflammatory profile suggesting better functions and pharmacological efficacy (Figure 6). Hence, CDCA improved primary islet delivery and diabetes treatment. Future studies need to explore dose-response of CDCA and potentially other bile acids in tissue delivery, biotechnology, and transplantation.

## Data Availability

The data presented in this study are available on request from the corresponding author. The data are not publicly available due to author property agreements.
